# Mechanisims of asthma and allergic disease – 1074. Prebiotics in infants for prevention of allergic disease and hypersensitiivity

**DOI:** 10.1186/1939-4551-6-S1-P71

**Published:** 2013-04-23

**Authors:** John Sinn

**Affiliations:** 1 Obstetrics, Gynaecology and Neonatology, University of Sydney, Sydney, Australia

## Background

Prebiotics (promote growth of ‘healthy’ bacteria) added to infant feeds have the potential to prevent sensitisation of infants to dietary allergens.

## Methods

Standard methods of the Cochrane Neonatal Review Group were used. Searches were updated October 2011. Randomised and quasi-randomised controlled trials that compared a prebiotic to control were eligible.

## Results

Three studies enrolling 1315 infants and reporting outcomes of 1126 infants (85%) were included. Several ongoing studies and completed studies with no reported allergy results were identified. Overall, one study reported a significant reduction in asthma (134 infants; RR 0.31, 95% CI 0.14, 0.96; RD -0.13, 95% CI -0.25, -0.01; NNT 7.7) but no significant difference in urticaria (134 infants, RR 0.15, 95%CI 0.02, 1.16) to 2 years. Meta-analysis of 3 studies found a significant reduction in infant eczema (1126 infants, RR 0.64, 95%CI 0.44, 0.94; RD -0.04, 95%CI -0.07, -0.01; NNT 25) although there was moderate heterogeneity between studies. In subgroup analysis, individual studies reported a significant reduction in asthma and eczema to 2 years - in infants at high risk of allergy fed an extensively hydrolysed formula; and a reduction in infant eczema in low risk infants fed a cow’s milk formula. The studies reporting reductions in asthma or eczema used GOS/FOS combinations, one with added acidic OS, at a concentration 0.8 g/100ml of oligosaccharide.

## Conclusions

Further evidence is required before a prebiotic can be routinely recommended for prevention of allergy. A well powered, independent trial is required to answer this question.

